# Emerging roles of circular RNAs in gastric cancer metastasis and drug resistance

**DOI:** 10.1186/s13046-022-02432-z

**Published:** 2022-07-11

**Authors:** Xiaolin Wang, Jiahui Zhang, Guozhen Cao, Jinghan Hua, Ge Shan, Wenchu Lin

**Affiliations:** 1 Department of Clinical Laboratory, The First Affiliated Hospital of USTC, Chinese Academy of Sciences (CAS) Key Laboratory of Innate Immunity and Chronic Disease, School of Basic Medical Sciences, Division of Life Science and Medicine, University of Science and Technology of China (UTSC), Hefei, 230027 Anhui China; 2 High Magnetic Field Laboratory, Hefei Institutes of Physical Science (HIPS), Chinese Academy of Sciences, Hefei, 230031 Anhui China; 3 University of Science and Technology of China, Hefei, 230027 Anhui China; 4 Sir Run-Run Shaw Hospital, Zhejiang University School of Medicine, Zhejiang, 310016 Hangzhou China; 5 Key Laboratory of High Magnetic Field and Ion Beam Physical Biology, HIPS, Chinese Academy of Sciences, Hefei, 230031 Anhui China

**Keywords:** circRNA, Gastric cancer, Metastasis, miRNA sponge, RNA binding protein, Drug resistance

## Abstract

Gastric cancer (GC) is an aggressive malignancy with a high mortality rate and poor prognosis, primarily caused by metastatic lesions. Improved understanding of GC metastasis at the molecular level yields meaningful insights into potential biomarkers and therapeutic targets. Covalently closed circular RNAs (circRNAs) have emerged as crucial regulators in diverse human cancers including GC. Furthermore, accumulating evidence has demonstrated that circRNAs exhibit the dysregulated patterns in GC and have emerged as crucial regulators in GC invasion and metastasis. However, systematic knowledge regarding the involvement of circRNAs in metastatic GC remains obscure. In this review, we outline the functional circRNAs related to GC metastasis and drug resistance and discuss their underlying mechanisms, providing a comprehensive delineation of circRNA functions on metastatic GC and shedding new light on future therapeutic interventions for GC metastases.

## Background

Gastric cancer (GC) is an aggressive and heterogeneous malignancy [1, 2]. With a median overall survival (OS) of 16 months among all patients, GC remains the fourth leading cause of cancer-related mortality worldwide [1–3]. Metastasis is a crucial process characterized by increased invasion and the ability of cancer to spread from its site of origin to other regions of the body, accounting for 90% of cancer-related deaths [4, 5]. Most GC patients are diagnosed at advanced stages and are frequently accompanied by invasion and metastasis, such as lymph node and peritoneum metastases [6, 7]. In metastatic (late) GC patients, the clinical outcomes are extremely poor, while the 5-year overall survival rate of early GC patients can reach over 90% [4, 7]. In addition, metastatic GC has long been considered less effective for surgical treatment and more resistant to drug therapy [8, 9]. Up to date, no effective methods or approaches are applied to treat metastatic GC [8, 9]. Recently, significant advances have been made in clarifying GC metastasis [5, 10]; however, the overall delineation of the molecular mechanisms is limited and ambiguous. Therefore, an in-depth understanding of GC metastasis at the molecular and cellular levels is imperative to identify potential biomarkers for diagnosis and therapeutic targets for intervention.

**Fig. 1:**
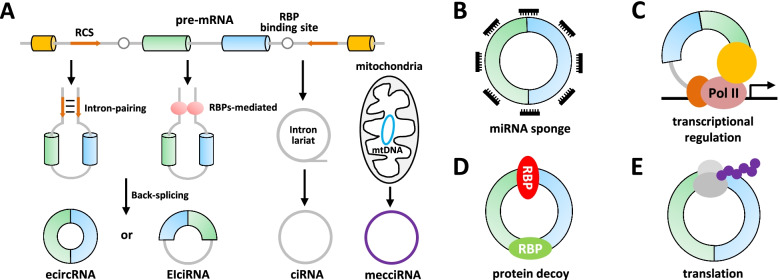
Biogenesis and function of circRNAs. **A**. The flanking introns of circularized exons contain reverse complementary sequences, which form a circular structure through direct base pairing, or RBP binding sites, which generate a circular structure via RBPs dimerization. The introns are removed or retained to form exonic circRNA (ecircRNA) or exon-intron circRNA (EIciRNA). Intronic circRNA (ciRNA) is derived from an intron by preventing intron debranching after splicing. Mitochondria encoded circRNAs (mecciRNAs) probably circularize via a splicing-independent mechanism. **B**. CircRNAs serving as miRNA sponges. **C**. CircRNAs promoting the transcription of targets through interacting with proteins or complexes such as U1 snRNP. **D**. CircRNAs behaving as protein decoys. **E**. CircRNAs working via generating polypeptides

Covalently closed circular RNAs (circRNAs) are single-stranded endogenous RNA molecules with loop structures and are resistant to exonuclease activity [11–13]. The biogenesis of circRNAs is widely acknowledged via a back-splicing event from precursor RNA (pre-RNA), which is facilitated by the flanking reverse complementary sequences, such as *Alu* elements, and is regulated by some RNA binding proteins (RBPs), including QKI, DHX9, FUS, Sam68, hnRNP L, hnRNPM and ADARs (Fig. 1A) [14–23].

Thousands of circRNAs across species have been identified and characterized through high-throughput sequencing combined with bioinformatic analyses in the past decade [24, 25]. Most circRNAs are chiefly derived from known protein-coding genes, consist of a single or multiple exon(s) (exonic circRNAs, ecircRNAs), and generally localize to the cytoplasm [13]. The most prominent function of cytoplasmic ecircRNAs is to serve as competing endogenous RNAs (ceRNAs) or miRNA sponges to lift the inhibitory effects of miRNAs on their downstream targets (Fig. 1B) [24, 26–28]. Interestingly, the intronic sequences between the circularized exons may be retained, forming exon-intron circRNAs (EIciRNAs) [29]. EIciRNAs are proved to enhance their parental gene expressions *in cis* via binding to the U1 small nuclear ribonucleoprotein (snRNP) complex in the nucleus (Fig. 1C) [29]. Intronic lariat precursors escaping from debranching produce intronic circRNAs (ciRNAs), which could regulate RNA polymerase II (Pol II)-mediated transcription in the nucleus [30, 31]. Besides, circRNAs directly interact with RBPs to regulate key targets as protein scaffolds or antagonists in various biological processes as well (Fig. 1D) [32–34]. In addition, a small fraction of ecircRNAs undergoes cap-independent translation to encode small peptides through the internal ribosome entry site (IRES)-driven mechanisms, although the vast majority of circRNAs are thought to be non-coding RNAs (Fig. 1E) [35–37]. Recently, a novel class of circRNAs encoded by mitochondria (mecciRNAs) has been reported to facilitate the mitochondrial entry of nuclear-encoded proteins by serving as molecular chaperones [38].

Accumulating evidence has pointed out the aberrant expression patterns of circRNAs and their regulatory roles in cancer progression and metastasis [39–44]. Systematic and comprehensive knowledge regarding circRNAs related to GC metastasis expands our understanding of the underlying mechanisms of metastatic GC. In the present review, we overview the current research status of circRNAs in GC metastasis, including modulating epithelial-mesenchymal transition (EMT), regulating angiogenesis, exosomal circRNAs, and drug resistance, and discuss the potential clinical application value of circRNAs in GC. We hope to provide insights into circRNAs-mediated GC metastasis and their potential as putative biomarkers or therapeutic targets of GC in the future.

## CircRNAs participate in EMT

EMT, a highly complex and dynamic process, is recognized as a vital step driving the early phase of cancer metastasis [45, 46]. Recently, several circRNAs have been reported to participate in EMT by modulating various signaling pathways, such as TGF-β/SMAD, Wnt/β-catenin, and PI3K/AKT pathways [47]; thereby, we summarized up-to-date information on circRNAs engaged in these signaling pathways in GC metastasis (Table 1).

**Table 1:** A list of circRNAs related to GC metastasis

CircRNA	CircBase ID	Expression	Property in metastasis	Molecular mechanism	Refs
*circTHBS1*	*hsa_circ_0034536*	Up	Enhancer	Modulate the *miR-204-5p/*INHBA axis and interact with the RBP, HuR	[48]
*circCCDC66*	*hsa_circ_0001313*	Up	Enhancer	Activate c-Myc/TGF-β signaling pathway	[49]
*circ_0001829*	*hsa_circ_0001829*	Up	Enhancer	Sponge *miR-155-5p* to upregulate SMAD	[50]
*circOXCT1*	*hsa_circ_0004873*	Down	Repressor	Sponge *miR-136* to upregulate SMAD4	[51]
*circAXIN1*	*hsa_circ_0005838*	Up	Enhancer	Encode a novel protein, AXIN1-295aa	[52]
*circFGD4*	*hsa_circ_0000390*	Down	Repressor	Sponge *miR-532-3p* to upregulate APC	[53]
*circREPS2*	*hsa_circ_0139996*	Down	Repressor	Sponge *miR-558* to upregulate RUNX3	[54]
*circAKT3*	*hsa_circ_0000199*	Up	Enhancer	Sponge *miR-198* to upregulate PIK3R1	[55]
*circ_0023409*	*hsa_circ_0023409*	Up	Enhancer	Sponge *miR-542-3p* to upregulate IRS4	[56]
*ciRS-7*	*hsa_circ_0001946*	Up	Enhancer	Sponge *miR-7* to upregulate PTEN	[57]
*circTNPO3*	*hsa_circ_0001741*	Down	Repressor	Interact with the RBP, IGF2BP3	[58]
*circFNDC3B*	*hsa_circ_0006156*	Up	Enhancer	Interact with the RBP, IGF2BP3	[59]
*circ_100876*	*hsa_circ_0023404*	Up	Enhancer	Sponge *miR-665* to upregulate YAP1	[60]
*circPRRX1*	*hsa_circ_0004370*	Up	Enhancer	Sponge *miR-665* to upregulate YWHAZ	[61]
*circRanGAP1*	*hsa_circ_0063526*	Up	Enhancer	Regulate the *miR-877-3p/*VEGFA axis	[62]
*circ_0044366*	*hsa_circ_0044366*	Up	Enhancer	Sponge *miR-29a* to upregulate VEGF	[63]
*circURI1*	*hsa_circ_0000921*	Up	Repressor	Interact with the splicing factor hnRNPM	[64]
*ebv-circLMP2A*	*-*	Up	Enhancer	Form a positive feedback loop with HIF1α	[65]
*circNRIP1*	*hsa_circ_0004771*	Up	Enhancer	Sponge *miR-149-5p* to upregulate AKT1	[66]
*circNEK9*	*hsa_circ_0032683*	Up	Enhancer	Sponge *miR-409-3p* to upregulate MAP7	[67]
*circRELL1*	*hsa_circ_0001400*	Down	Repressor	Sponge *miR-637* to upregulate EPHB3	[68]
*circSHKBP1*	*hsa_circ_0000936*	Up	Enhancer	Modulate the *miR-582-3p*/HuR/VEGF axis and interact with HSP90	[69]
*circMRPS35*	*hsa_circ_0000384*	Down	Repressor	Recruit the histone modifier, KAT7	[70]
*circMAPK1*	*hsa_circ_0004872*	Down	Repressor	Encode a MAPK1-109aa protein	[71]
*circRPL15*	*hsa_circ_0064574*	Up	Enhancer	Sponge *miR-502-3p* to upregulate OLFM4	[72]
*circUBE2Q2*	*hsa_circ_0005151*	Up	Enhancer	Modulate the *miR-370-3p*/STAT3 axis	[73]
*circAGO2*	*hsa_circ_0135889*	Up	Enhancer	Interact with the RBP, HuR	[74]
*circHuR*	*hsa_circ_0049027*	Down	Repressor	Transcriptionally repression *in cis*	[75]

### TGF-β/SMAD signaling pathway

**Fig. 2:**
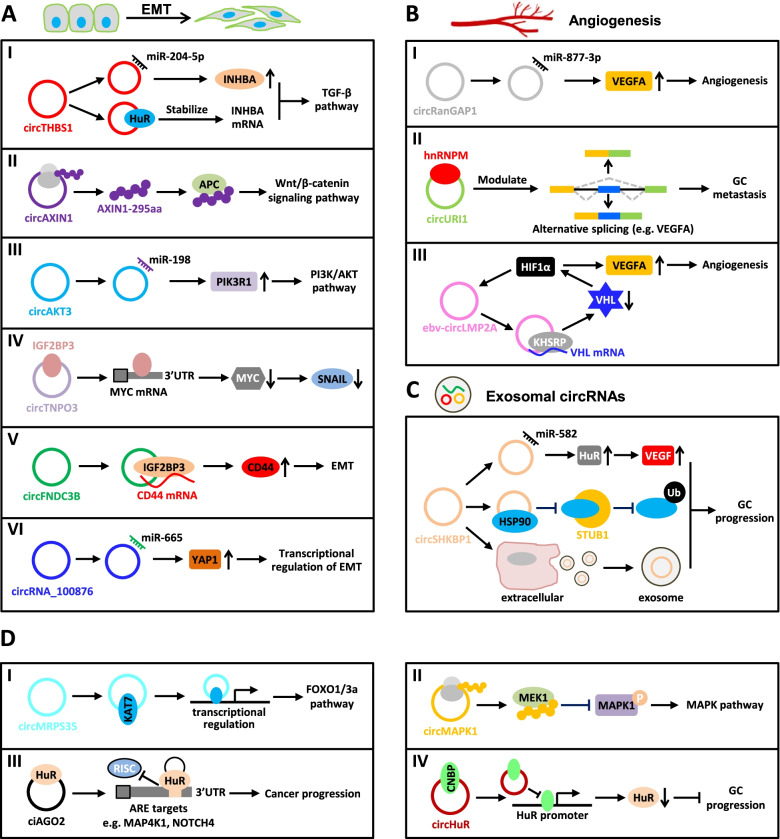
Molecular mechanisms of circRNAs related to GC metastasis. **A**. Roles of circRNAs in signaling pathways associated with EMT. I. The circRNA *circTHBS1* increases the INHBA level via adsorbing *miR-204-5p* in a sponge form, and stabilizes the *INHBA* mRNA via sequestering HuR protein, leading to the activation of the TGF-β pathway. II. *CircAXIN1*-encoded a novel protein, AXIN1-295aa interacts with APC to activate the canonical Wnt/β-catenin signaling pathway. III. The circRNA *circAKT3* activates the PI3K/AKT signaling by serving as a ceRNA against *miR-198* to upregulate PIK3R1. IV. *CircTNPO3* competitively binds to IGF2BP3, leading to the destabilization of the *MYC* mRNA, consequently repressing the expressions of MYC and its target SNAIL. V. The *circFNDC3B* level is significantly increased in GC and *circFNDC3B* interacts with IGF2BP3 protein and *CD44* mRNA to form a ternary complex, resulting in the upregulation of CD44, which facilitates EMT in GC. VI. The circRNA *circ_100876* interacts with *miR-665* to relieve the repressive effect on its target YAP1, which is involved in the transcriptional activation of EMT-related genes. **B**. Roles of circRNAs engaged in angiogenesis. I. *CircRanGAP1* is validated to stimulate angiogenesis via modulating the *miR-877-3*/VEGFA axis. II. *CircURI1*, a highly expressed circRNA in GC, sequesters the splicing factor, hnRNPM protein in a sequence-dependent manner to modulate alternative splicing of a subset of migration-related genes, such as VEGFA, consequently inhibiting GC metastasis. III. *Ebv-circLMP2A* promotes angiogenesis through forming a positive feedback loop with HIF1α to improve the VEGFA expression. Under hypoxia, HIF1α up-regulates *ebv-circLMP2A*, and *ebv-circLMP2A* interacts with KHSRP to destabilize the VHL mRNA, resulting in VHL down-regulation and HIF1α accumulation. **C**. Exosomal circRNA in GC. The circRNA *circSHKBP1* promotes GC progression via the *miR-582-3p*/HuR/VEGF axis, and sequestering HSP90 to suppress STUB1-mediated HSP90 ubiquitination. Additionally, increased exosomal *circSHKBP1* could facilitate co-cultured cell growth. **D**. Other pivotal pathways or targets involved in GC metastasis. I. The circRNA *circMRPS35* inhibits GC tumorigenesis through the recruitment of histone acetyltransferase KAT7 to the promoters of FOXO1/3a genes, activating the FOXO1/3a transcription, consequently triggering the FOXO1/3a pathway. II. The circRNA *circMAPK1* exerted an anti-tumor effect on GC invasion via generating a 109aa protein forming as a molecular sponge for MEK1, thus inhibiting the phosphorylation of MAPK1 and eventually leading to the inactivation of the MAPK pathway. III. The ciRNA *circAGO2* interacts with HuR protein to promote its activation and enrichment on the 3’ UTR of HuR targets, resulting in repressing the AGO2/miRNA-mediated gene silencing involved in cancer progression. IV. The circRNA *circHuR* sequesters CNBP from the HuR’s promoter, leading to the repressions of HuR and GC progression

The TGF-β/SMAD signaling is a classic pathway in cancer metastasis [47]. The circRNA *circTHBS1*, which is highly expressed in GC and associated with poor prognosis, is reported to promote the malignant behaviors and EMT of GC cells by triggering the INHBA/TGF-β pathway [48]. Mechanistically, *circTHBS1* behaves as a *miR-204-5p* sponge to enhance the INHBA expression, and it also stabilizes the *INHBA* mRNA mediated by HuR, consequently activating the TGF-β pathway (Fig. 2AI) [48]. The *circCCDC66* expression is elevated in GC and related to tumor stage and lymphatic metastasis [49]. Gain- and loss-of-function studies have revealed that *circCCDC66* promotes GC metastasis by activating c-Myc and the TGF-β signaling pathways [49]. In another case, *hsa_circ_0001829* promotes GC cell migration and invasion *in vitro* and GC metastasis *in vivo* via modulating the *miR-155-5p*/SMAD axis [50]. A similar ceRNA mechanism also applies to *circOXCT1*, which interacts with *miR-136* to relieve the repressive effect on its target SMAD4, inhibiting GC EMT and metastasis [51].

### Wnt/β-catenin signaling pathway

The Wnt/β-catenin signaling pathway is indispensable among the pathways regulated by circRNAs in EMT [47, 52–54]. The *circAXIN1* expression is significantly up-regulated in GC compared to the corresponding non-tumor gastric tissues [52]. Silencing of *circAXIN1* suppresses GC cell proliferation, migration, and invasion, whereas the ectopic expression of *circAXIN1* promotes GC malignancy *in vitro* and *in vivo* [52]. Mechanistically, a novel protein AXIN1-295aa encoded by *circAXIN1* competes with parental AXIN1 protein to bind APC and release β-catenin, consequently activating the canonical Wnt/β-catenin signaling pathway to facilitate GC progression (Fig. 2AII) [52]. Additionally, Dai et al. have proposed that the *circFGD4* expression is markedly attenuated in GC tissues and negatively correlated with lymphatic metastasis and the short prognosis of GC patients [53]. Furthermore, *circFGD4* shows its anti-tumor effect on GC tumorigenesis and metastasis by modulating the *miR-532-3p*/APC/β-catenin axis [53]. Similarly, *circREPS2* exhibits a decreased level in GC and inhibits GC migration and invasion via repression of the RUNX3/β-catenin pathway by sequestering *miR-558* [54].

### PI3K/AKT signaling pathway

The PI3K/AKT signaling pathway is frequently activated in EMT during metastasis and a series of dysregulated circRNAs have been found to interfere with this pathway [47, 55–57]. For example, GC-specific *circAKT3* activates the PI3K/AKT signaling by repressing miR-198-mediated inhibition of PIK3R1, a regulatory subunit of PI3K (Fig. 2AIII) [55]. The circRNA *hsa_circ_0023409* is highly expressed in GC tissues and markedly correlated with tumor size, histological grade, and TNM staging, nominating it as a potential prognostic marker for GC [56]. Functionally, *hsa_circ_0023409* exerts the oncogenic effects on GC progression and metastasis by competitively sponging *miR-542-3p* to enhance the expression of IRS4, which contributes to activating the PI3K/AKT pathway [56]. A well-characterized circRNA, *CDR1as* (*ciRS-7*), is markedly up-regulated in GC and linked to poor survival in an independent validation cohort, and promotes GC cell migration and metastasis via antagonizing the *miR-7*-mediated expression of PTEN, which is broadly regarded as a negative regulator of the PI3K/AKT signaling pathway [57, 76].

### Other pathways

Several additional circRNAs have been gradually characterized to engage in other EMT signaling pathways [58–61]. For example, *circTNPO3* is significantly downregulated in GC compared with matched noncancerous tissues and plasma *circTNPO3* owns the ability to serve as a potential diagnostic biomarker [58]. *In vitro* and *in vivo* observations reveal that *circTNPO3* suppresses GC proliferation and metastasis [58]. Mechanistically, *circTNPO3* competitively interacts with IGF2BP3 and subsequently destabilizes the *MYC* mRNA, ultimately inhibiting MYC and its target SNAIL, a primary and key inducer of EMT (Fig. 2AIV) [58]. The circRNA *circFNDC3B* appears to be increased in GC significantly and facilitates cell migration, invasion and EMT of GC cells by forming a ternary complex of *circFNDC3B*-IGF2BP3-CD44 mRNA (Fig. 2AV) [59]. In addition, *circ_100876*, a significantly up-regulated circRNA in GC, contributes to GC migration and invasion by serving as a molecular sponge for *miR-665* to regulate the expression of YAP1, which activates a transcriptional program involved in EMT (Fig. 2AVI) [60].

Collectively, these findings strongly indicate that circRNAs can modify several critical biological pathways relevant to GC metastasis.

## CircRNAs regulate angiogenesis

Angiogenesis, defined as the formation of new blood vessels sprouting from preexisting vessels, is well-regarded as an important initial step in cancer metastasis [77–79]. Several signaling pathways, including VEGFA and HIF1α signaling, can continuously induce angiogenesis, aggravating cancer progression [80, 81]. Recently, several circRNAs have been reported to participate in GC metastasis by regulating VEGFA- or HIF1α-mediated angiogenesis [62–65].

The circRNA *circRanGAP1* is validated to sponge *miR-877-3p* to increase the VEGFA expression, stimulate angiogenesis and promote GC metastasis (Fig. 2BI) [62]. A similar ceRNA mechanism also applies to *circ_0044366*, which binds to *miR-29a* to derepress the VEGF expression and thus facilitates angiogenesis and migration in GC [63]. The circRNA *circURI1* back-spliced from exons 3–4 of URI1 has been identified from circRNA profiling of 5 paired GC and adjacent non-cancerous (paraGC) specimens [64]. *CircURI1* exhibits a remarkably higher expression in GC than paraGC tissues and is negatively associated with metastasis in GC patients [64]. Functional studies perform that *circURI1* inhibits GC metastasis *in vitro* and *in vivo*. Mechanistically, *circURI1* behaved as a decoy of hnRNPM in a sequence-dependent manner to modulate alternative splicing of a subset of genes related to cell migration, thus suppressing GC metastasis (Fig. 2BII) [64]. VEGFA is a direct and functional target of *circURI1*, and *circURI1* can promote exon 7 inclusion of VEGFA (VEGFA^e7IN^) [64]. *CircURI1*-induced VEGFA^e7IN^ possesses a greater ability to prevent the *circURI1*-silencing-mediated promoting effect on GC cell invasion than exon 7 exclusion of VEGFA [64, 82]. This study firstly reported the engagement of circRNA-modulated alternative splicing in cancer metastasis [64].

Additionally, virus-encoded circRNA has also been found to engage in angiogenesis in GC [65, 83]. Epstein-Barr virus (EBV)-derived circRNA LMP2A (*ebv-circLMP2A*) is correlated with distant metastasis and poor prognosis in EBV-associated GC (EBVaGC) [65]. Furthermore, the *ebv-circLMP2A* expression is positively correlated with the expressions of HIF1α and VEGF in clinical samples of EBVaGC and a mouse model [65]. Ectopic expression of *ebv-circLMP2A* promotes angiogenesis and GC cell migration under hypoxia, while *ebv-circLMP2A* knockdown reverses these effects [65]. Mechanistic studies reveal that HIF1α and *ebv-circLMP2A* form a positive feedback loop, which promotes angiogenesis in EBVaGC [65]. Briefly, under hypoxia, HIF1α induces the *ebv-circLMP2A* expression, and *ebv-circLMP2A* interacts with KHSRP to enhance the VHL mRNA decoy mediated by KHSRP, resulting in HIF1α accumulation (Fig. 2BIII) [65].

## Exosomal circRNAs and GC metastasis

Exosomes are small extracellular vesicles with an average diameter of ~100 nanometers, containing an abundant cargo of proteins and different RNA species, including circRNAs, which can enhance substance exchange between cells and improve signal transduction [84, 85]. Accumulating evidence has demonstrated that exosomes play emerging roles in regulating cancer metastasis and treatment through the transfer and exchange of molecules during cell-cell communications [86, 87]. Recently, circRNAs have been shown to be abundant in exosomes and exosomal circRNAs might be regarded as circulating biomarkers for metastatic disease in GC patients [88, 89].

Multiple exosomal circRNAs from the plasmas of GC patients are involved in GC invasion and metastasis [66–69]. *CircNRIP1* possesses a significantly higher expression level in exosomes from GC plasma than in normal tissues and engages in exosomal crosstalk between GC cells [66]. GC cells co-cultured with exosomes derived from *circNRIP1*-overexpressed cells exhibit higher metastatic potential than control cells via the tail vein metastasis model [66]. Simultaneously, exosomal *circNRIP1* promotes GC metastasis *in vivo* and regulates EMT by activating the AKT1/mTOR signaling pathway via sponging *miR-149-5p* [66]. Similarly, *circNEK9,* an up-regulated circRNA in GC tissues, accelerates GC proliferation by serving as a ceRNA against *miR-409-3p* to target MAP7 [67]. Additionally, the exosome-mediated transfer of *circNEK9* performs promotive effects on GC cell migration and invasion [67]. Sang et al. have uncovered that exosomal *circRELL1* is down-regulated in GC, and its delivery mediated by GC cells-derived exosomes stimulate autophagy by modulating the *miR-637*/EPHB3 axis in GC progression [68]. In another case, *circSHKBP1* is remarkably upregulated in both GC tissues and serum and is significantly associated with advanced TNM stage and poor survival [69]. Mechanistically, exosomal *circSHKBP1* promotes GC cell migration and invasion via modulating the *miR-582-3p*/HuR/VEGF axis, and inhibiting HSP90 ubiquitination through sequestering HSP90 to obstruct its interaction with STUB1 (Fig. 2C) [69]. These promising results provide novel insights into therapy and the predictions of GC prognosis.

## Other metastasis-related pivotal pathways or targets

### FOXO1/3a pathway

The FOXO1/3a pathway stimulates the expressions of the downstream targets, including p21, p27, Twist1, and E-cadherin [70, 90]. The circRNA *circMRPS35* is identified from circRNA profiles of three paired GC and the corresponding non-tumor tissues, whose level is associated with clinicopathological characteristics and prognosis in GC patients [70]. Biologically, *in vivo* observations and *in vitro* experiments reveal that *circMRPS35* inhibits GC cell proliferation and invasion [70]. Furthermore, mechanistic studies reveal that *circMRPS35* combats GC tumorigenesis by recruiting histone acetyltransferase KAT7 to transcriptionally activate the FOXO1/3a genes, consequently triggering the FOXO1/3a pathway (Fig. 2DI) [70].

### MEK-MAPK pathway

The MEK-MAPK signaling pathway is mainly involved in GC proliferation and metastasis [71, 91]. The circRNA *circMAPK1* exhibits a decreased level in GC compared to the corresponding adjacent non-tumor tissues and is inversely correlated with GC tumor size, lymphatic invasion, TNM stage, and poor OS [71]. Functional investigations implicate that *circMAPK1* suppresses GC proliferation and invasion *in vitro* and *in vivo* [71]. Mechanistically, *circMAPK1* exerts the anti-tumor effect through encoding a MAPK1-109aa protein as a molecular sponge for MEK1, thus suppressing the phosphorylation of MAPK1 and eventually resulting in the inactivation of the MAPK pathway (Fig. 2DII) [71].

### STAT3 pathway

Signal transducer and activator of transcription 3 (STAT3) is a widely-characterized oncogene in diverse human cancers [92, 93]. The circRNA *circRPL15*, up-regulated in GC tissues and correlated with short survival, enhances GC cell migration and invasion, and inhibits apoptosis by sequestering *miR-502-3p* from the OLFM4 mRNA to activate the STAT3 pathway [72]. A similar ceRNA mechanism also applies to *circUBE2Q2*, which interacts with *miR-370-3p* to relieve the inhibitory effect on its target STAT3 in GC, promoting proliferation, glycolysis, and metastasis [73].

### Human antigen R

Human antigen R (HuR), a classic RBP, is frequently up-regulated in multiple human cancers including GC and plays a vital role in cancer progression and metastasis [94]. An intronic circRNA *circAGO2* generated from the first intron of AGO2 is increased in GC and boosts GC metastasis *in vitro* and *in vivo* [74]. Mechanistic studies reveal that *circAGO2* physically interacts with HuR protein to facilitate its activation and enrichment on the 3’ UTR of HuR targets, inhibiting AGO2/miRNA-mediated gene silencing associated with cancer progression (Fig. 2DIII) [74]. In another case, *circHuR*, predominantly localized in the nucleus, is downregulated in GC tissues and suppresses GC cell growth, invasion, and metastasis [75]. Mechanistically, *circHuR* interacts with CNBP and subsequently restrains its binding to the promoter of HuR, leading to the repressions of HuR and GC progression (Fig. 2DIV) [75].

## Interplay between circRNAs and drug resistance in GC

Although chemo- and radio-therapy are recognized as the most effective and extensive treatment methods for GC patients after surgery during the past few decades, the clinical applications are still limited owing to the intrinsic and acquired resistance, resulting in the occurrence of distant metastasis in GC patients [1, 3, 95]. Additionally, targeted therapy and immunotherapy with immune checkpoint inhibitors for GC have emerged [96]. Convincing evidence has confirmed that diverse circRNAs influence drug resistance in GC therapeutic responses (Table 2) [55, 112].

**Table 2:** CircRNAs involved in drug resistance in GC

CircRNA	CircBase ID	Drug	Expression	Drug resistance	Targets	Refs
*circVAPA*	*hsa_circ_0006990*	Cisplatin	Up	Enhance	*miR-125b-3p*, STAT3	[97]
*circAKT3*	*hsa_circ_0000199*	Cisplatin	Up	Enhance	*miR-198*, PIK3R1	[55]
*circARVCF*	*hsa_circ_0092330*	Cisplatin	Up	Enhance	*miR-1205*, FGFR1	[98]
*circCCDC6*	*hsa_circ_0001313*	Cisplatin	Up	Enhance	*miR-618,* BCL-2	[99]
*circFN1*	*hsa_circ_0058147*	Cisplatin	Up	Enhance	*miR-182-5p*	[100]
*circPVT1*	*-*	Cisplatin	Up	Enhance	*miR-30a-5p,* YAP1	[101]
*circ_0000260*	*hsa_circ_0000260*	Cisplatin	Up	Enhance	*miR-129-5p,* MMP11	[102]
*circ_0032821*	*hsa_circ_0032821*	Oxaliplatin	Up	Enhance	*miR-515-5p*, SOX9	[103]
*circPVT1*	*-*	Paclitaxel	Up	Enhance	*miR-124-3p,* ZEB1	[104]
*circNRIP1*	*hsa_circ_0004771*	5-fluorouracil	Up	Enhance	*miR-138-5p*, HIF-1α	[105]
*circDLG1*	*hsa_circ_0008583*	anti-PD-1	Up	Enhance	*miR-141-3p*, CXCL12	[106]
*circCUL2*	*hsa_circ_0000234*	Cisplatin	Down	Suppress	*miR-142-3p*, ROCK2	[107]
*circMCTP2*	*hsa_circ_0000657*	Cisplatin	Down	Suppress	*miR-99a-5p*, MTMR3	[108]
*circ_0000144*	*hsa_circ_0000144*	Oxaliplatin	Down	Suppress	*miR-502-5p*, ADAM9	[109]
*circ_0000376*	*hsa_circ_0000376*	Bupivacaine	Down	Suppress	*miR-145-5p*	[110]
*circ_0000520*	*hsa_circ_0000520*	Herceptin	Down	Suppress	PI3K-AKT pathway	[111]

Cisplatin (CDDP) is one of the most effective chemotherapeutic agents for patients with GC, especially those in advanced stages [113, 114]. The *circVAPA* expression is elevated in CDDP-resistant GC cells, and *circVAPA* facilitates GC cell migration, invasion, and CDDP resistance [97]. Further mechanistic investigations indicate that *circVAPA* exerts its oncogenic activity through sponging with *miR-125b-5p* to increase the STAT3 expression [97]. Similarly, several other circRNAs such as *circAKT3*, *circPVT1*, *circFN1*, and *circ_0000260*, also enhance CDDP resistance and malignant progression in GC [55, 98–102]. Oxaliplatin (OXA) is a widely used anti-cancer medicine [115]. The circRNA *circ_0032821* is significantly increased in OXA-resistant GC cells and their derived exosomes, and contributes to OXA resistance, GC cell migration and invasion through derepressing SOX9 via sequestering *miR-515-5p* [103]. Paclitaxel (PTX) is an effective first-line chemotherapy drug in GC treatment, and *circPVT1* contributes to PTX resistance and GC cell invasion via serving as a ceRNA against *miR-124-3p* to target ZEB1, a crucial transcriptional inhibitor of E-cadherin [104]. 5-fluorouracil (5-FU) is currently a first-line agent for the clinical treatment of GC, and *circNRIP1* promotes hypoxia-induced 5-FU resistance via modulating the miR-138-5p/HIF-1α axis in GC [105]. Anti-programmed cell death protein 1 (PD-1) monoclonal antibody is a commonly used immune-checkpoint blockade agent for GC immunotherapy [116]. The circRNA *circDLG1* facilitates GC progression and anti-PD-1 resistance via *miR-141-3p*-mediated the regulation of CXCL12 [106].

On the other hand, various circRNAs reverse drug resistance in GC treatment [107–109]. Peng et al. have unveiled that *circCUL2* displays a decreased level in GC tissues and possesses a repressively regulatory function in CDDP resistance, GC cell migration, and invasion via *miR-142-3p*/ROCK2-mediated autophagy activation [107]. Another circRNA *circMCTP2* is reported to inhibit CDDP resistance of GC cells via the *miR-99a-5p*/MTMR3 axis [108]. The circRNA *hsa_circ_0000144* exerts inhibitory effects on OXA resistance, GC cell proliferation, and metastasis through up-regulating ADAM9 mediated by *miR-502-5p* [109]. Bupivacaine, a local anesthetic commonly used in the resection operation of GC patients, reduces the *circ_0000376* level in GC cells, and *circ_0000376* partially reverses bupivacaine-mediated repressive effects on GC cell viability and metastasis via sponging *miR-145-5p* [110]. Herceptin, a targeted therapy drug, is a humanized monoclonal antibody specifically binding to HER2 and acts as an antitumor role in GC [117]. The circRNA *hsa_circ_0000520* is significantly reduced in GC and reverses the Herceptin resistance of GC cells by inhibiting the PI3K/AKT signaling pathway [111].

Taken together, these studies provide the possibility that a combination of circRNAs-based therapy with chemotherapy, targeted therapy or immunotherapy may be a valuable approach to overcome drug resistance and prevent metastasis in GC in the future.

## Clinical significance of circRNAs in GC

CircRNAs have multiple remarkable characteristics which provide tremendous potential for serving as biomarkers and therapeutic targets owing to the covalently closed-loop structure, disease-specific and dynamic expression pattern and high conservation across species [118–122]. For example, according to a study by Liang and colleagues, *hsa_circ_0110389* has been identified as a diagnostic/prognostic biomarker and therapeutic target for GC [123]. Similarly, *circOSBPL10* might serve as a novel proliferation factor and prognostic marker of the OS and disease-free survival (DFS) of GC patients [124]. In another case, Chen et al. have displayed that the *circPVT1* level is an independent prognostic biomarker for OS and DFS in GC patients [125].

Since exosomes can be detected in various body fluids, including plasma, saliva, urine, and cerebrospinal fluid, exosomal circRNAs might be ideal noninvasive biomarkers for the diagnosis and/or prognosis of gastric cancer [88, 126]. For instance, the *circSHKBP1* expression is significantly increased in GC serum and positively correlated with advanced TNM stage and poor survival [69]. Furthermore, GC cell exosomes enhance co-cultured cell growth by delivering *circSHKBP1* [69]. These findings indicate that *circSHKBP1* is a promising circulating biomarker for GC diagnosis and prognosis [69]. Additionally, the circRNA *circRanGAP1* exhibits a significantly higher expression in plasma exosomes derived from GC patients than the healthy controls. It promotes GC cell migration and invasion, indicating that plasma exosomal *circRanGAP1* might serve as a promising biomarker for GC patients [62]. The circRNAs that show potential as biomarkers in GC are summarized in Table 3.

**Table 3:** Clinical significance of circRNAs in GC (Cases more than 50)

CircRNA	CircBase ID	Sample	Expression	Clinicopathologic Features	Prognosis	Refs
*circTHBS1*	*hsa_circ_0034536*	Tissue	Up	Size, stage, grade, LNM	OS	[48]
*circCCDC66*	*hsa_circ_0001313*	Tissue	Up	Stage, LNM	-	[49]
*circOXCT1*	*hsa_circ_0004873*	Tissue	Down	Stage, LNM	OS	[51]
*circAXIN1*	*hsa_circ_0005838*	Tissue	Up	Stage, grade, LNM	-	[52]
*circFGD4*	*hsa_circ_0000390*	Tissue	Down	Grade, LNM	OS	[53]
*circREPS2*	*hsa_circ_0139996*	Tissue	Down	Size, stage, grade	-	[54]
*circAKT3*	*hsa_circ_0000199*	Tissue	Up	Size, stage, grade, chemoresistance	OS	[55]
*circ_0023409*	*hsa_circ_0023409*	Tissue	Up	Size, stage, grade	OS	[56]
*ciRS-7*	*hsa_circ_0001946*	Tissue	Up	Stage, LNM	OS	[57]
*circTNPO3*	*hsa_circ_0001741*	Tissue, plasma	Down	Differentiation	-	[58]
*circ_100876*	*hsa_circ_0023404*	Tissue	Up	Stage, LNM, BVI, LVI	DFS	[60]
*circRanGAP1*	*hsa_circ_0063526*	Tissue, plasma	Up	Size, stage, LNM	OS	[62]
*circURI1*	*hsa_circ_0000921*	Tissue	Up	Stage, tumor metastasis	-	[64]
*ebv-circLMP2A*	-	Tissue	Up	Stage, LNM, tumor metastasis	OS, DFS	[65]
*circNRIP1*	*hsa_circ_0004771*	Tissue	Up	Size, LNM	OS, DFS	[66]
*circRELL1*	*hsa_circ_0001400*	Tissue, plasma	Down	Stage, LNM, differentiation	OS, DFS	[68]
*circSHKBP1*	*hsa_circ_0000936*	Tissue	Up	Size, stage, vascular invasion	OS	[69]
*circMRPS35*	*hsa_circ_0000384*	Tissue	Down	Size, stage, LNM	OS	[70]
*circMAPK1*	*hsa_circ_0004872*	Tissue	Down	Size, stage, LNM	OS	[71]
*circUBE2Q2*	*hsa_circ_0005151*	Tissue, plasma	Up	Size, lymphatic invasion	-	[73]
*circAGO2*	*hsa_circ_0135889*	Tissue	Up	-	OS	[74]
*circHuR*	*hsa_circ_0049027*	Tissue	Down	Stage, tumor metastasis	OS	[75]
*circVAPA*	*hsa_circ_0006990*	Tissue	Up	-	*-*	[97]
*circFN1*	*hsa_circ_0058147*	Tissue	Up	Stage, grade, chemoresistance	*-*	[100]
*circCUL2*	*hsa_circ_0000234*	Tissue	Down	Stage, LNM, differentiation	OS	[107]
*circMCTP2*	*hsa_circ_0000657*	Tissue	Down	Size, stage, grade, chemoresistance	OS, DFS	[108]
*circ_0110389*	*hsa_circ_0110389*	Tissue	Up	Stage, differentiation	OS, DFS	[123]
*circOSBPL10*	*hsa_circ_0008549*	Tissue	Up	Stage, grade	OS, DFS	[124]
*circPVT1*	*-*	Tissue	Up	Stage, nervous invasion	OS, DFS	[125]

## Conclusions and future perspectives

Current active research in circRNAs has brought us a range of exciting findings implying that circRNAs are of great importance in various diseases [11, 118, 127–129]. A tremendous amount of evidence has demonstrated the abnormal expression pattern of circRNAs in GC and the involvement of circRNAs in GC metastasis and drug resistance [11, 64, 126]. We have systematically described a series of dysregulated circRNAs in GC and elucidated their underlying molecular mechanisms in GC metastasis and drug resistance (Tables 1 and 2).

To date, various circRNA candidates have been validated and engaged in GC metastasis based on a series of molecular and cellular experiments [64, 66–69, 124, 125]. However, a global and comprehensive understanding of circRNAs related to GC metastasis is still scarce. To gain better and deeper insight into the aberrant expression pattern of circRNAs involved in GC metastasis, genome-wide circRNA profiling with high throughput sequencing from metastatic and non-metastatic GC tissues is a powerful approach to address this issue.

Four subclasses of circRNAs have been identified, including ecircRNAs, EIciRNAs, ciRNAs and mitochondria-encoded circRNAs (mecciRNAs) [11, 38, 130]. Current literature about circRNAs in GC metastasis generally includes ecircRNAs and ciRNAs, their functions and the molecular mechanisms [72–75, 94, 126]. Nevertheless, two other kinds of circRNAs and their functions have not been evaluated, which presents an exciting field to explore further.

The well-characterized mechanism of circRNAs is to sequester miRNAs to regulate the expressions of targeted genes [11–13]. A single circRNA could function as a scaffold for several different miRNAs [123]. Conversely, a miRNA can target multiple circRNAs as well [60, 61]. Identification and construction of the circRNA-miRNA regulatory network will help to systematically decipher the roles of circRNAs in GC metastasis in the future. In addition to the ceRNA mechanism, circRNAs have various molecular modes of action, including participating in epigenetic regulations, modulating alternative splicing, and generating protein [64, 71, 75]. We expect a burst of circRNA studies to elucidate some novel mechanisms of action in GC metastasis in the upcoming years.

Considering that circRNAs possess unique features such as tissue- and developmental stage-specific patterns, structural resistance to exonucleases and longer half-lives, and specific circRNAs play essential roles in GC metastasis and drug resistance, manipulating circRNA abundance appears to be a promising therapeutic strategy for the advanced GC treatment [126–128, 131, 132]. Furthermore, combining circRNAs-based therapeutic interventions with traditional chemotherapy or targeted therapy offers a unique opportunity to conquer drug resistance in advanced GC patients [97–111, 113–117]. However, choosing crucial target circRNAs of interest is still a problem. Furthermore, precisely and effectively delivering circRNAs into targeted cells for tumor treatment is also a significant issue that needs to be solved.

## Conclusions

In summary, the advances in circRNAs research will be essential to unravel their potential significance in GC. Furthermore, a better understanding of the association between circRNAs and GC would make circRNAs promising candidates as valuable biomarkers or potential targets in GC treatment.

## Data Availability

All data generated or analyzed during this study are included in this published article.

## Abbreviations

5-FU: 5-fluorouracil
BVI: Blood vessel infiltration
CDDP: Cisplatin
ceRNA: Competing endogenous RNA
circRNA: Circular RNA
ciRNA: Intronic circRNA
DFS: Disease-free survival
ecircRNA: exonic circRNA
EIciRNA: exon-intron circRNA
EMT: Epithelial-mesenchymal transition
EBV: Epstein-Barr virus
EBVaGC: EBV-associated GC
GC: Gastric cancer
HuR: Human antigen R
IRES: Internal ribosome entry site
LNM: Lymph node metastasis
LVI: Lymphatic vessel infiltration
mecciRNA: mitochondria-encoded circRNA
OS: Overall survival
OXA: Oxaliplatin
paraGC: adjacent non-cancerous GC
PD-1: Programmed cell death protein 1
Pol II: Polymerase II
pre-RNA: precursor RNA
PTX: Paclitaxel
RBP: RNA binding protein
snRNP: small nuclear ribonucleoprotein
STAT3: Signal transducer and activator of transcription 3
VEGFA^e7IN^: Exon 7 inclusion of VEGFA
